# A Synthetic Disaccharide Derivative of Diphyllin, TAARD, Activates Human Natural Killer Cells to Secrete Interferon-Gamma *via* Toll-Like Receptor-Mediated NF-κB and STAT3 Signaling Pathways

**DOI:** 10.3389/fimmu.2018.01509

**Published:** 2018-07-18

**Authors:** Long Yi, Luxi Chen, Xiaofeng Guo, Ting Lu, Haixia Wang, Xiaotian Ji, Jianying Zhang, Yulin Ren, Pan Pan, A. Douglas Kinghorn, Xiaohua Huang, Li-Shu Wang, Zhijin Fan, Michael A. Caligiuri, Jianhua Yu

**Affiliations:** ^1^Research Center for Nutrition and Food Safety and Third Affiliated Hospital, Third Military Medical University, Chongqing, China; ^2^The Ohio State University Comprehensive Cancer Center, Columbus, OH, United States; ^3^Biomedical Sciences Graduate Program, Medical Scientist Training Program, The Ohio State University, Columbus, OH, United States; ^4^State Key Laboratory of Elemento-Organic Chemistry, College of Chemistry, Nankai University, Tianjin, China; ^5^Center for Biostatistics, Department of Bioinformatics, The Ohio State University, Columbus, OH, United States; ^6^Division of Medicinal Chemistry and Pharmacognosy, College of Pharmacy, The Ohio State University, Columbus, OH, United States; ^7^Division of Hematology and Oncology, Medical College of Wisconsin, Milwaukee, WI, United States; ^8^Department of Chemistry, The University of Memphis, Memphis, TN, United States; ^9^Division of Hematology, Department of Internal Medicine, College of Medicine, The Ohio State University, Columbus, OH, United States; ^10^The James Cancer Hospital, Columbus, OH, United States

**Keywords:** natural killer cells, natural products, interferon gamma, diphyllin, immunotherapy

## Abstract

Natural products and their derivatives have long been used as pharmacological agents in the fight against cancer. Human natural killer (NK) cells are critical in our immune system in that they are capable of destroying tumor cells directly. However, there are few reports that elucidate the role of natural products in activating NK cells. In this study, we discovered that a synthetic disaccharide derivative of diphyllin, 4-*O*-{[2′′,3′′,4′′-tri-*O*-acetyl-α-D-arabinopyranosyl-(1′′→4′)]-2′,3′-di-*O*-acetyl-α-L-rhamnopyranosyl}diphyllin (TAARD), can alone stimulate interferon (IFN)-γ secretion in primary human NK cells and the NKL cell line. Additionally, it had an additive effect with IL-12 or IL-15 on IFN-γ production, but little adverse effects on NK cells. Mechanistically, TAARD induced the phosphorylation of NF-κB and STAT3, resulting in their binding on the *IFNG* promoter, which was dependent on TLR1 and TLR3 signaling, respectively. STAT3 and NF-κB knockdown with lentivirus shRNA as well as the NF-κB-specific inhibitor, *N*-tosyl-l-phenylalaninechloromethyl ketone, significantly suppressed TAARD-induced IFN-γ generation in primary NK cells. Blockade of TLR1 and TLR3 with neutralizing antibodies considerably decreased TAARD-induced activation of NF-κB and STAT3, respectively, as well as IFN-γ generation in NK cells. Collectively, our data suggest that TAARD can induce NK cell IFN-γ production through TLR1-NF-κB and TLR3-STAT3 signaling pathways, rendering its potential use as an agent for cancer prevention or treatment.

## Introduction

Since the early 1800s, natural products have played a crucial role in drug discovery and the development of new therapies for the treatment of human disease. In fact, over 50% of all current oncologic therapies are directly derived from natural products and their related structures (1). Currently, many new naturally occurring compounds and their derivatives are undergoing clinical trials, predominantly as agents in fighting cancer and infection (2). Drugs derived from natural sources have been used in a wide range of therapeutic areas, including metabolism, hormone regulation, inflammation, cardiovascular disease, and neurological conditions. With the advent of cutting-edge technology, new natural products will continue to be developed as better treatments for cancer to improve human health (3–5).

The innate immune system serves as an immediate first line defense mechanism against invading pathogens and tumor cells (6). Natural killer (NK) cells are one of the predominant mediators of this process, possessing both cytokine-producing and cytotoxicity functions (7). These large granular lymphocytes provide the body’s major source of interferon-gamma (IFN-γ), a cytokine that is highly important in activating macrophages to kill obligate intracellular pathogens, shaping the immune response of Th1 helper cells, activating antigen presenting cells to enhance upregulation of MHC Class I, and eradicating malignant tumor cells and viruses (8). Indeed, decreased secretion of IFN-γ by NK cells has been linked to an increased incidence of infection and malignancy (9). However, exogenously delivered IFN-γ has been studied in several clinical trials with little success due to its toxic effects (10). Efforts to augment endogenous IFN-γ production *via* cytokines such as IL-12, IL-15, or IL-2 have also been tried (11) but present with limitations, including systemic toxicity due to activation of multiple immune effector cells (12) and T regulatory cell induction (13).

*Phyllanthus* (Phyllanthaceae) is a large genus with over 700 species that produce lignans as one of their major groups of secondary metabolites. In our search for natural NK cell stimulators, a phytosterol characterized from the aerial parts of *Phyllanthus songboiensis* was found to be active (14), and several phyllanthusmins have been identified as potent and selective cytotoxic agents from different parts of *Phyllanthus poilanei* (15), while phyllanthusmin C (PL-C) has been reported for its enhancement of IFN-γ production by human NK cells through upregulation of toll-like receptor (TLR)-mediated NF-κB signaling (16). Additionally, diphyllin glycoside justiprocumin B was reported to have potent activity against a broad spectrum of HIV strains with an IC_50_ of 15–21 nM (17). Following these investigations, we generated a synthetic disaccharide derivative of diphyllin, 4-*O*-{[2′′,3′′,4′′-tri-*O*-acetyl-α-d-arabinopyranosyl-(1′′→4′)]-2′,3′-di-*O*-acetyl-α-L-rhamnopyranosyl} diphyllin, or TAARD, and evaluated the NK cell stimulatory effect of TAARD. The results showed that this compound increases the secretion of IFN-γ by activating NK cells through TLR-mediated NF-κB and STAT3 signaling pathways. This finding promotes a new avenue for anticancer drug discovery through the modification of natural products to enhance innate immune responses to cancer or virally infected cells.

## Materials and Methods

### Chemicals

A derivative of the natural product diphyllin, totally acetylated arabinopyranosyl rhamnopyranosyl diphyllin, TAARD (4-*O*-{[2′′,3′′,4′′-tri-*O*-acetyl-α-d-arabinopyranosyl-(1′′→4′)]-2′,3′-di-*O*-acetyl-α-l-rhamnopyranosyl}diphyllin) [Figure S1 in Supplementary Material, C_42_H_44_O_20_ (MW 868.79), purity > 99%] was synthesized.

### Enrichment, Purification, and Sorting of Primary Human NK Cells

Primary human NK cells were isolated directly from fresh peripheral blood leukopaks (American Red Cross, Columbus, OH, USA) as described previously (18). The Ohio State University Institutional Review Board approved all human subject work. NK cells (CD56^+^CD3^−^) were enriched with the NK Cell Isolation Kit (Miltenyi Biotec) according to the manufacturer’s protocol with minor modifications. Enriched NK cells were further purified by positive selection using anti-CD56 magnetic-activated cell sorting beads (Miltenyi Biotec). The purity of enriched and purified NK cells were >70% (Figure S2A in Supplementary Material) and >98% (Figure S2B in Supplementary Material), respectively, with assessment by flow cytometric analysis after staining with CD56-allophycocyanin (APC) and CD3-fluorescein isothiocyanate (FITC) Abs (BD Biosciences). Pure (>99.0%) CD56^bright^ and CD56^dim^ NK cell populations were isolated by cell sorting (FACS Aria II, BD Biosciences).

### Cell Culture and Treatment

Primary NK cells and the NKL cell line were cultured in RPMI 1640 medium (Invitrogen), supplemented with 50 µg/ml penicillin, 50 µg/ml streptomycin, and 20% FBS (Invitrogen) at 37°C in 5% CO_2_. The NKL cell line was cultured with 150 IU/mL recombinant human IL-2 (Hoffman-LaRoche), but cells were starved of IL-2 for 24 h prior to stimulation. Cells were treated with different concentrations (0.1, 1, and 10 µM) of TAARD in the presence or absence of IL-12 (10 ng/mL) or IL-15 (100 or 10 ng/mL) (R&D Systems) for 18 h. To detect protein phosphorylation by immunoblotting, cells were treated for 6 h prior to protein extraction. To study NF-κB involvement in TAARD-mediated enhancement of NK cell IFN-γ production, 10 µM of the NF-κB inhibitor, *N*-tosyl-l-phenylalaninechloromethyl ketone (TPCK), was used to pretreat both purified primary NK cells and NKL cells for 1 h prior to TAARD treatment. For TLR blocking assays, NK cells were pretreated with 10 µg/mL of an anti-human TLR-1 antibody (InvivoGen), an anti-human TLR-3 antibody (Hycult Biotech) (19), an anti-human TLR-6 antibody (InvivoGen) (20), or various combinations for 1 h prior to stimulation with TAARD. Cells treated with the same concentration of non-specific rat and mouse IgG antibodies were used as controls. The blocking antibodies were also kept in culture medium during stimulation.

### Proliferation Assay

Cell counting and the 3-(4,5-dimethylthiazol-2-yl)-5-(3-carboxymethoxyphenyl)-2-(4-sulfophenyl)-2*H*-tetrazolium (MTS) assay were used to determine the cellular proliferation of primary human NK cells. Briefly, purified NK cells were cultured in 96-well plates and treated with different concentrations (0.1, 1, and 10 µM) of TAARD. NK cells cultured alone without stimulation were used as a control group. The number of cells was recorded every 2 days. Simultaneously, the absorbance was measured spectrophotometrically at 490 nm every 3 days using a BioTek plate reader (BioTek, UK).

### Flow Cytometric Evaluation of Apoptosis

Purified NK cells were treated with different concentrations (0.1, 1, and 10 µM) of TAARD. Following washing with PBS, cells were resuspended in 100 µL of 1 × annexin-V binding buffer and incubated with 5 µL of an annexin V-FITC antibody (PharMingen) for 15 min at room temperature in the dark. Next, 1 mL of 1× annexin-V binding buffer was added and centrifuged, followed by resuspension of the cells in 200 µL 1× annexin-V binding buffer. Then, 5 µL of 7-amino-actinomycin D (7-AAD) was added, and cells were incubated for 5 min at room temperature in the dark. Cells were first washed with 500 µL of 1× annexin-V binding buffer and then resuspended in 200 µL of 1× annexin-V binding buffer, followed by filtration through a 70-µm nylon filter (Corning). The samples were placed on ice and quantitatively analyzed by a FACS flow cytometer.

### Enzyme-Linked Immunosorbent Assay (ELISA)

Primary human NK or NKL cells were plated in 96-well plates (Corning Costar, Catalog #CLS3595). After treatment, cell-free supernatants were collected to determine IFN-γ secretion by ELISA with commercially available mAb pairs (Thermo Fisher Scientific), according to the manufacturer’s protocol.

### Real-Time Reverse Transcription Polymerase Chain Reaction (RT-PCR)

Real-time RT-PCR was performed as described previously (21, 22). Briefly, total RNA from purified primary human NK cells or NKL cells was extracted with an RNeasy Mini Columns (Qiagen). cDNA synthesis was performed with random primers (Invitrogen). mRNA expression levels of *IFNG, TLR1, TLR3, TLR6, NF-*κ*B/p65, I*κ*B*α, *STAT3, STAT4, STAT5a*, and *STAT5b* were detected by SYBR Green Master Mix (Thermo Fisher Scientific) on the Applied Biosystems ViiA 7 Real-time PCR system (Life Technologies). The primers used are shown in Table S1 of the Supplementary Material. The relative expression ratio was normalized to the *HPRT1* internal control and analyzed by the ΔΔCt method.

### Immunoblotting

Immunoblotting was performed as described previously (21, 22). Cells were collected, re-suspended in RIPA lysis buffer (23) containing protease/phosphatase inhibitors, and incubated on ice for 30 min. Then, the protein lysate was mixed with 4× Laemmli buffer (Bio-Rad, Catalog #1610747) supplemented with 2.5% 2-Mercaptoethanol (2-ME), boiled for 5 min, and subjected to immunoblotting analysis. Abs against p-STAT3, STAT3, p-STAT4, STAT4, p-STAT5, STAT5, pNF-κB/p65, and NF-κB/p65 (1:1,000 dilution, Cell Signaling Technology) were used for immunoblotting. An antibody against β-actin (Santa Cruz Biotechnology) was utilized as an internal control. The proteins were visualized using enhanced chemiluminescence (GE Healthcare Life Sciences) reagents.

### Electroporation, Transfection, and Luciferase Reporter Assay

Activation of TLR1-NF-κB and TLR3-STAT signaling pathways was assessed in the NKL cell line and the human embryonic kidney 293T cell line *via* a luciferase reporter assay. To introduce plasmids into NKL cells through electroporation, NKL cells were washed and resuspended in Opti-MEM medium (Thermo Fisher Scientific) at a concentration of 1.0 × 10^7^ cells ml^−1^. Cells were mixed with a pGL3-κB-Luc (3-κB; 1 µg) (16), 4×M67 pTATA TK-Luc (4xM67; 1 µg; Addgene), or pGL3-Basic (1 µg; Promega) plasmid and a pRL-TK renilla-luciferase control plasmid (50 ng; Promega). The 3-κB plasmid contains three tandem repeats of the κB site. The 4×M67 plasmid has four copies of GG**TTCCCGTAA**ATGCATCA, in which **TTCCCGTAA** is the STAT binding site (24). Subsequently, cells were transferred into 4 mm electroporation cuvettes (Biorad, Hercules). Electroporation was performed using a Super Electroporator NEPA 21 (Nepa gene) under the following conditions: 275 V, 2.0 ms pulse length, and 50 ms pulse interval. After electroporation, cells were immediately transferred into fresh media and cultured for an additional 6 h prior to TAARD treatment. Then, 293T cells were co-transfected with lipofectamine 2000 containing the plasmid 3-κB, 4×M67, or pGL3-Basic (1 µg each) with pRL-TK (5 ng), with or without TLR1, TLR3, or TLR6 expression plasmids (0.5 µg each) for 24 h. After electroporation or co-transfection, both NKL and 293T cells were then treated with various concentrations of TAARD for an additional 24 h. Thereafter, cells were lysed and both firefly and renilla luciferase activities were measured sequentially with the Dual-Glo Luciferase Reporter Assay System (Promega) according to the manufacturer’s instructions. The ratio of firefly/renilla luciferase activities was used to determine the relative activities of NF-κB or STAT.

### Knockdown of STAT3 Using Lentivirus Short Hairpin RNA (shRNA) in NKL Cells

Lentiviral-mediated shRNA was used to permanently knockdown *STAT3* expression in NKL cells. To create lentiviral particles, we established the stable NKL cell line expressing *STAT3* shRNA or scramble shRNA using lentiviral infection (pSIH1-puro-STAT3 shRNA, Addgene #26596; pSIH1-puro-control shRNA, Addgene #26597). This was followed by drug selection with puromycin. The target sequence for *STAT3* was CATCTGCCTAGATCGGCTA (25). Lentiviral vectors were produced by transient transfection of shRNA plasmids along with packaging plasmids into 293T cells. Briefly, a total of 5 × 10^6^ cells were seeded in 75-cm^2^ tissue culture flasks 24 h before transfection. Cells were supplied with fresh DMEM medium 2 h prior to transfection. 20 µg of the lentiviral vector was mixed with 10 µg of the VSV-G envelope plasmid and 15 µg of the packaging plasmid (pCMVDR9). The solution was mixed with 0.5 M CaCl_2_ and adjusted to 1 mL with water, then mixed with 1 mL of 2 × HEPES-buffered saline (280 mM NaCl, 10 mM KCl, 1.5 mM Na2HPO_4_, 12 mM dextrose, 50 mM HEPES, pH 7.2). Two milliliters of the vector solution was added drop-wise directly to the cells. The medium was replaced after 16 h, and the vector-containing supernatants were harvested 48 h after transfection. After filtering through a 0.45-µm-pore-size filter, the supernatants were then spun at 25,000 × *g* for 2 h in a Beckman ultracentrifuge Optima XL-100k with a 28-Ti fixed-angle rotor. After centrifugation, the viral pellets were re-suspended in 600 µL of PBS and stored at −80°C. Following that, NKL cells were cultured in the concentrated virus-containing medium and centrifuged at 1,800 rpm at 32°C for 1.5 h. Cells were allowed to recover for 24 h and then supplemented with puromycin (1 mg/mL) for 72 h to select for cells that had stably incorporated into the vector. Cells were harvested and knockdown of *STAT3* by lentiviral shRNA was confirmed by immunoblotting.

### Statistical Analysis

Unpaired Student’s *t* tests were used to compare two independent conditions (i.e., TAARD versus control) for continuous endpoints. Paired *t* tests were used to compare two conditions with repeated measures from the same donor. ELISA data were transformed for statistical analysis by log base 2 due to its skewed distribution. A one-way ANOVA model was used for multiple comparisons. A two-way ANOVA model was used to evaluate the synergistic or additive effect between IL-12 or IL-15 with TAARD. The *p* values were adjusted for multiple comparisons using the Bonferroni method. All tests are two-sided. A *p* value of 0.05 was considered statistically significant.

## Results

### TAARD Does not Affect Proliferation and Survival of Primary Human NK Cells

We first determined whether TAARD affected cell proliferation and survival of human NK cells. Our results showed that treatment with different concentrations (0.1, 1, and 10 µM) of TAARD had little effects on cell proliferation, as determined by counting cultured NK cells in the presence or absence of TAARD (Figure S3A in Supplementary Material) and an MTS assay (Figure S3B in Supplementary Material). TAARD also did not significantly affect the survival of NK cells, as determined by an annexin V plus 7-AAD flow cytometric assay (Figures S3C,D in Supplementary Material). This suggests that TAARD likely does not have adverse effects on primary human NK cells.

### TAARD Significantly Increases IFN-γ Production in NK Cells

**Figure 1:**
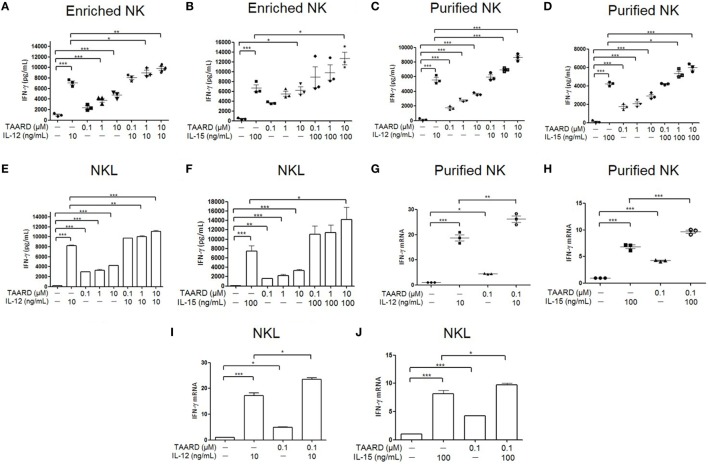
TAARD stimulates interferon (IFN)-γ production in natural killer (NK) cells. **(A,B)** Enriched human NK cells were treated with DMSO vehicle control or different concentrations (0.1, 1, and 10 µM) of TAARD for 18 h in the presence or absence of IL-12 (10 ng/mL) **(A)** or IL-15 (100 ng/mL) **(B)**. Cell-free supernatants were collected and analyzed by enzyme-linked immunosorbent assay (ELISA) to determine IFN-γ release in enriched human NK cells (*n* = 3 in each treatment). **(C,D)** Highly purified (>98%) human NK cells were treated with DMSO vehicle control or different concentrations (0.1, 1, and 10 µM) of TAARD for 18 h in the presence or absence of IL-12 (10 ng/mL) **(C)** or IL-15 (100 ng/mL) **(D)** to determine the level of IFN-γ release by ELISA. **(E,F)** NKL cells were treated with DMSO vehicle control or various concentrations (0.1, 1, and 10 µM) of TAARD for 18 h in the presence or absence of IL-12 (10 ng/mL) **(E)** or IL-15 (100 ng/mL) **(F)**. Cell-free supernatants were collected and analyzed by ELISA to determine IFN-γ release in enriched NK cells. **(G,H)** Highly purified NK cells were treated with DMSO vehicle control or 0.1 µM of TAARD for 18 h in the presence or absence of IL-12 (10 ng/mL) **(G)** or IL-15 (100 ng/mL) **(H)**. *IFNG* mRNA expression was assessed by real-time RT-PCR and relative *IFNG* mRNA expression for each treatment group was normalized to the untreated vehicle control in the same donor. **(I,J)** NKL cells were treated with DMSO vehicle control or 0.1 µM of TAARD for 18 h in the presence or absence of IL-12 (10 ng/mL) **(I)** or IL-15 (100 ng/mL) **(J)**. *IFNG* mRNA expression was assessed by real-time RT-PCR and relative *IFNG* mRNA expression of each treatment group was normalized to the untreated vehicle control. The data are presented as mean ± SEM (*n* = 3 for each treatment; error bars represent the SEM for primary NK cells). Data shown as mean ± SD for NKL cells, which represent at least three independent experiments. *p < 0.05, **p < 0.01, and ***p < 0.001 denote statistical comparison between the two marked treatment groups.

Human NK cells were treated with various concentrations of TAARD alone or in the presence of IL-12 (Figures 1A,C) or IL-15 (Figures 1B,D). IFN-γ secretion was determined *via* ELISA assays. Our data showed that in both enriched and purified NK cells, all three concentrations (0.1, 1, and 10 µM) of TAARD alone stimulated the production of higher levels of IFN-γ compared to untreated cells (Figures 1A–D). Moreover, the results indicated a dose-dependent effect, with 10 µM TAARD inducing the highest level of IFN-γ secretion from NK cells. We observed an additive effect in enriched as well as purified NK cells treated with 10 µM of TAARD and IL-12 or IL-15. Likewise, in the NKL cell line, we observed a similar response of NK cells to stimulation with TAARD alone (Figures 1E,F). Additionally, there is also an additive effect observed in NKL cells treated with TAARD (10 µM) and IL-12 or IL-15. We then measured transcriptional expression of the IFN-γ gene after treatment with TAARD alone and in combination with IL-12 or IL-15 in purified primary NK cells (Figures 1G,H) as well as NKL cells (Figures 1I,J). Even at a low concentration of 0.1 µM, TAARD alone was able to induce IFN-γ mRNA expression in both purified NK cells and NKL cells. Moreover, IFN-γ was also induced by TAARD (10 µM) in NK cells of five tested donors in the presence of the potent cytokine stimuli, the combination of IL-12 (5 ng/mL) with IL-15 (10 ng/mL) (data not shown). Collectively, these data suggest that TAARD has the capacity to stimulate IFN*-*γ production in NK cells.

### TAARD Augments IFN-γ Secretion in Both CD56^bright^ and CD56^dim^NK Subsets

**Figure 2:**
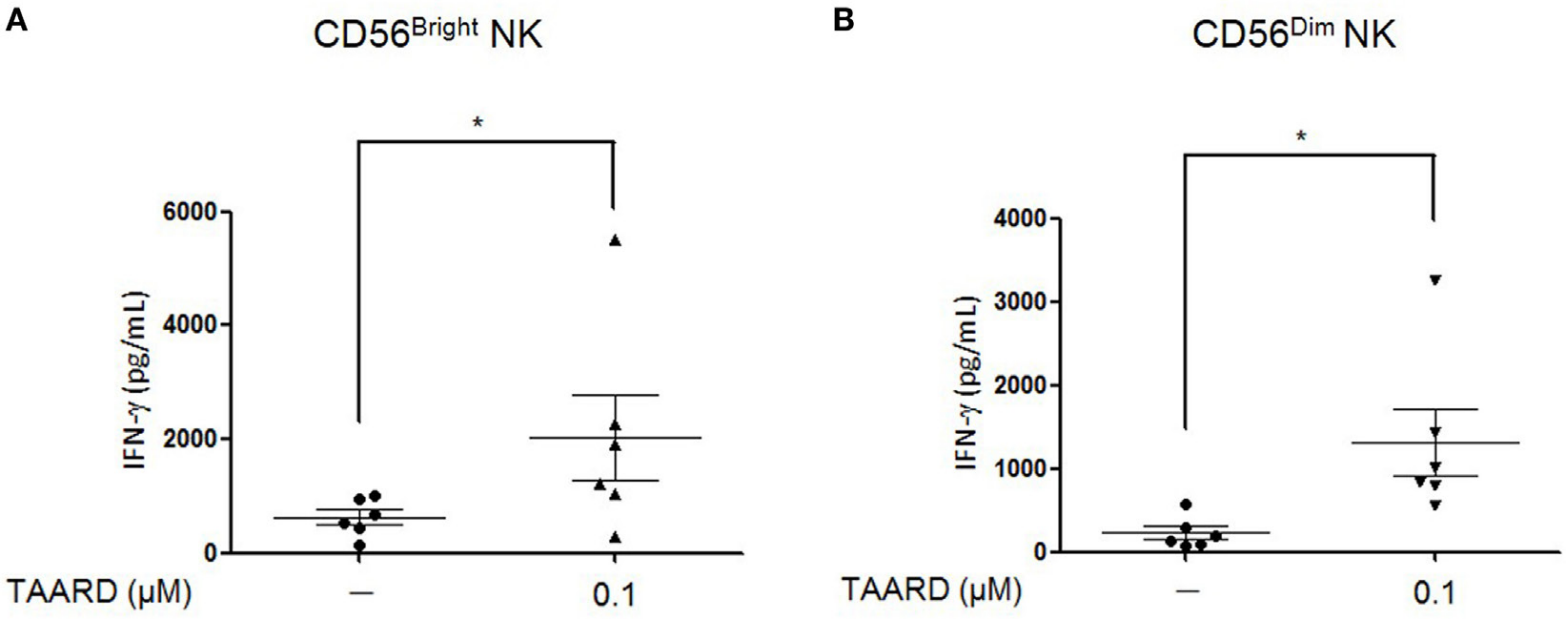
TAARD treatment augments interferon (IFN)-γ secretion by both CD56^bright^ and CD56^dim^ natural killer (NK) cells. Enriched NK cells were sorted *via* FACS into CD56^dim^ and CD56^bright^ NK cells, based on the relative density of CD56 expressed on the cell surface. **(A)** CD56^bright^ NK cells were treated with or without 0.1 µM of TAARD for 18 h in the absence of cytokine stimulation. IFN-γ secretion was determined by ELISA. **(B)** CD56^dim^ NK cells were treated and analyzed as shown in **(A)**. Data shown are collected from six donors. *p < 0.05 compared to the untreated group.

Human NK cells can be divided into two different subsets according to the surface density of the CD56 antigen. The CD56^dim^ population has a low-density expression of the CD56 antigen, while the CD56^bright^ subset has a high level expression (26). The majority, about 90%, of NK cells in the peripheral blood is composed of CD56^dim^ cells while the rest (10%) are CD56^bright^. The two human NK subsets were purified to 99.5% purity (Figures S4A,B in Supplementary Material). Here, we demonstrated that in both subsets of NK cells, 0.1 µM of TAARD alone was capable of significantly increasing IFN-γ secretion in the two populations compared to the vehicle control (Figures 2A,B).

### TAARD Facilitates the Phosphorylation of NF-κBp65 and STAT3 Proteins in NK Cells

**Figure 3:**
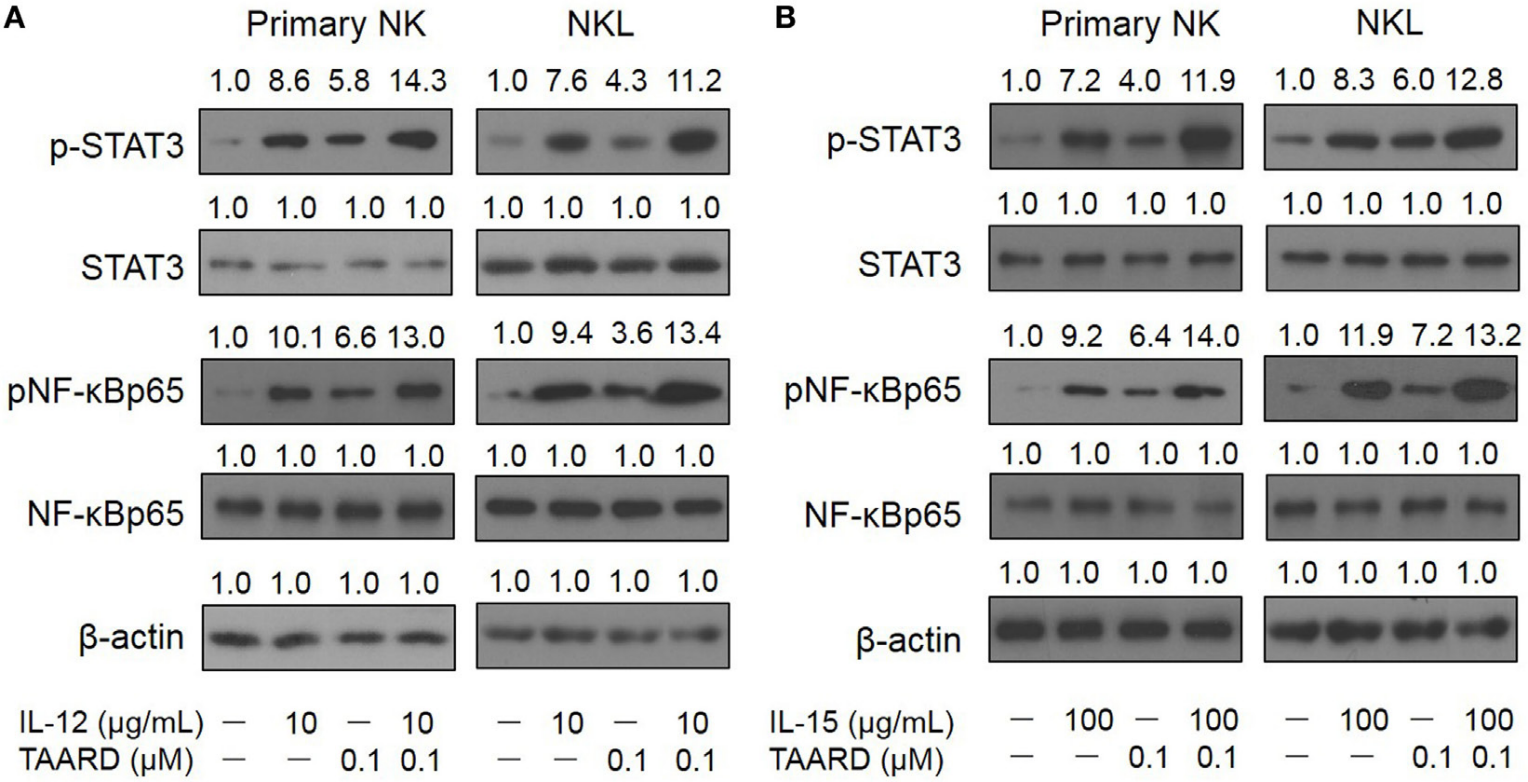
TAARD induces the phosphorylation of NF-κB and STAT3 in NK cells. **(A,B)** Purified primary human natural killer cells and NKL cells were treated with 0.1 µM of TAARD alone or in combination with 10 ng/mL of IL-12 **(A)** or 100 ng/mL of IL-15 **(B)** for 6 h. Cells were harvested and lysed for immunoblotting using their corresponding Abs. β-actin was included as internal control.

Transcriptional modulation of IFN-γ expression mainly involves JAK-STAT and NF-κB signaling pathways. To determine whether the induction of IFN-γ by TAARD in NK cells is correlated with these signaling pathways, we performed immunoblotting of protein lysates extracted from primary human NK cells as well as the NKL cell line. We found that 0.1 µM of TAARD alone increased the phosphorylation of p65, a subunit of the transcriptional complex NF-κB, and the phosphorylation of STAT3 in primary human NK cells and the NKL cell line (Figures 3A,B). We found that TAARD did not appear to have a significant effect on the phosphorylation of STAT4 or STAT5 proteins (Figures S5A,B in Supplementary Material). It appeared that there was at least an additive effect on the phosphorylation of p65 and STAT3 when TAARD was combined with IL-12 (Figure 3A) or IL-15 (Figure 3B). No changes were observed in total NF-κB or STAT3 protein when primary NK or NKL cells were treated with TAARD alone. No significant changes in the level of NF-κBp65/RelA, STAT3, STAT4, STAT5α, and STAT5b transcripts were observed either in primary NK and NKL cells treated with TAARD (Figures S6A,B in Supplementary Material). Therefore, the above data suggest that TAARD specifically facilitates the phosphorylation of NF-κBp65 and STAT3 in NK cells.

### TAARD Induces the Luciferase Reporter Activities Corresponding to NF-κB and STAT3 Activation *via* TLR1 and TLR3, Respectively

**Figure 4:**
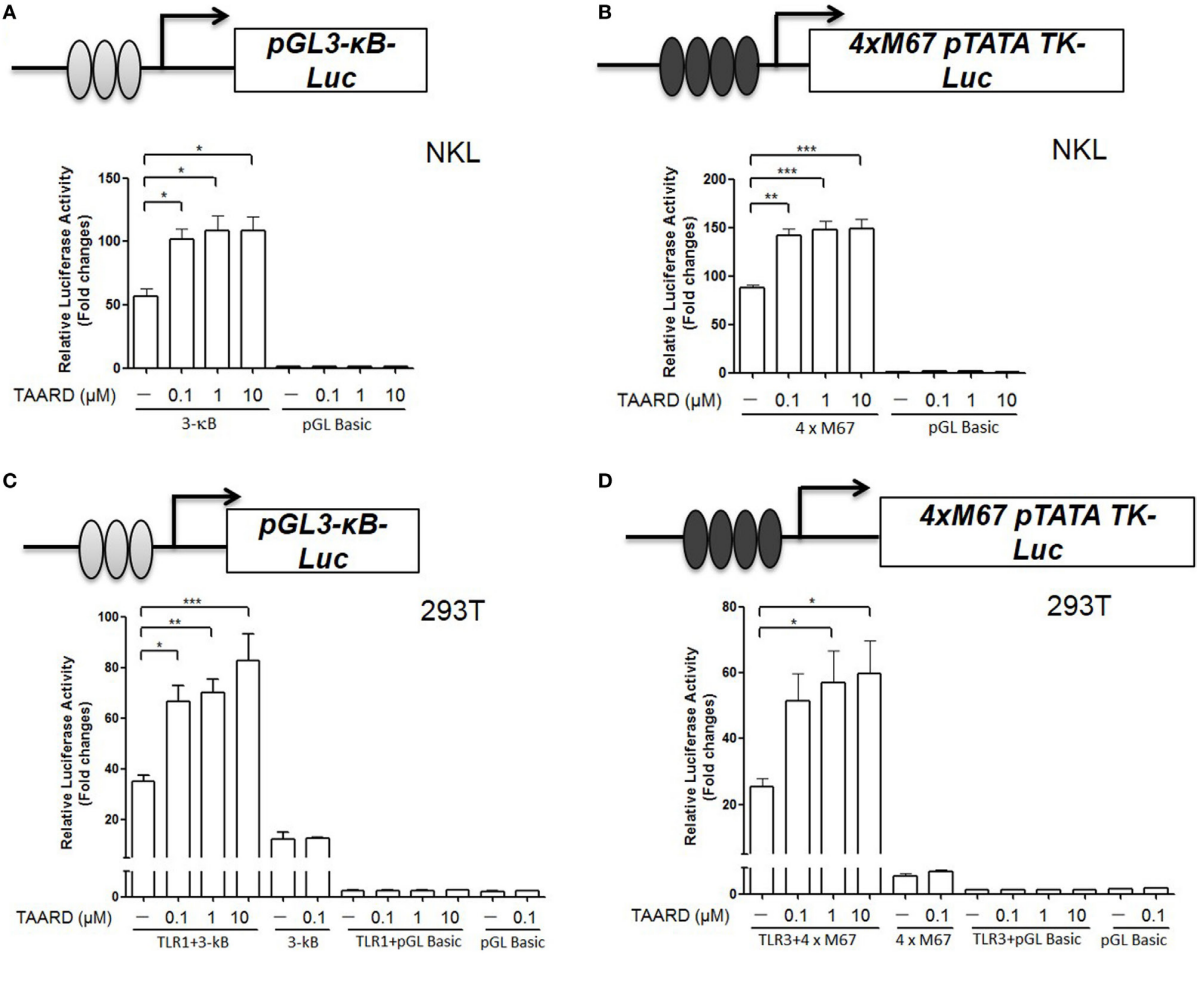
TAARD increases the luciferase reporter activities of NF-κB and STAT3 *via* TLR1 and TLR3, respectively. **(A)** NKL cells were electroporated with either pGL3-κB-Luc (1 µg) or pGL3-Basic (1 µg) and a pRL-TK renilla-luciferase control plasmid (50 ng). After electroporation, cells were immediately transferred into fresh medium and cultured for an additional 6 h. Then, cells were treated with different concentrations of TAARD. **(B)** NKL cells were electroporated as described in **(A)** but with 4×M67 pTATA TK-Luc plasmid (1 µg). Cells were treated and measured as described in **(A)**. **(C)** 293T cells were co-transfected with pGL3-κB-luc (1 µg) or pGL3-Basic (1 µg) and pRL-TK renilla-luciferase control plasmids (5 ng) in the presence or absence of a TLR1 (0.5 µg) expression plasmid by Lipofectamine 2000. Twenty-four hours later, cells were treated with different concentrations (0.1, 1, and 10 µM) of TAARD for another 24 h with fresh medium. **(D)** 293T cells were co-transfected with 4×M67 pTATA TK-Luc (1 µg) or pGL Basic plasmid (1 µg) and pRL-TK renilla-luciferase control plasmids (5 ng) in the presence or absence of a TLR3 (0.5 µg) expression plasmid by Lipofectamine 2000. Twenty-four hours later, cells were treated with different concentrations (0.1, 1, and 10 µM) of TAARD for another 24 h with fresh medium. The ratio of firefly to renilla luciferase activities was used to show the relative luciferase activity, which corresponded to NF-κB or STAT activation. Data shown represent one of three independent experiments with similar results. *p < 0.05, **p < 0.01, and ***p < 0.001 denote statistical comparison between the two marked treatment groups.

Toll-like receptor signaling is upstream of NF-κB and STAT3 signaling and activation of TLRs leads to cytokine gene expression in immune cells (27). TLR1, TLR3, and TLR6 are the main TLR receptors expressed on NK cells (18). Therefore, we investigated whether TAARD augmented *IFNG* transcription *via* TLR-mediated induction of NF-κB and STAT3 binding to their respective sites within the *IFNG* promoter. By a luciferase assay, we found that the promoter reporter activity increased significantly with all three concentrations of TAARD (0.1, 1, and 10 µM) when the 3-κB plasmid containing three tandem NF-κB (Figure 4A) or 4×M67 plasmid containing four tandem STAT binding sites (Figure 4B) were transfected in the NKL cell line. Additionally, our data demonstrated that after co-transfection with TLR1 and 3-κB in 293T cells, TAARD treatment induced a significantly increased NF-κB luciferase activity in the TLR1 and 3-κB co-transfected cells compared to the cells without TAARD treatment (Figure 4C). Likewise, relative luciferase activity increased significantly in TLR3 plus 4×M67 co-transfected 293T cells compared to the cells without TAARD treatment (Figure 4D). However, co-transfection of 293T cells with 3-κB plus TLR3 or TLR6 (Figures S7A,B in Supplementary Material) and 4×M67 plus TLR1 or TLR6 (Figures S7C,D in Supplementary Material) resulted in no significant changes in relative luciferase activity. It should be noted that 293T fibroblast cells lack most of the TLRs, and thus, TAARD could not induce luciferase activity in 293T cells without a TLR plasmid (Figures 4C,D) (28). These results imply that the increase of κB and STAT3 luciferase reporter activities is dependent on the specific activation of TLR1 and TLR3, respectively. This is consistent with previous studies showing that TLR1 and TLR3 result in enhanced activation of NF-κB and STAT signaling pathways (29).

### TLR1-NF-κB and TLR3-STAT3 Signaling Pathways Mediate NK Cell Activation by TAARD

**Figure 5:**
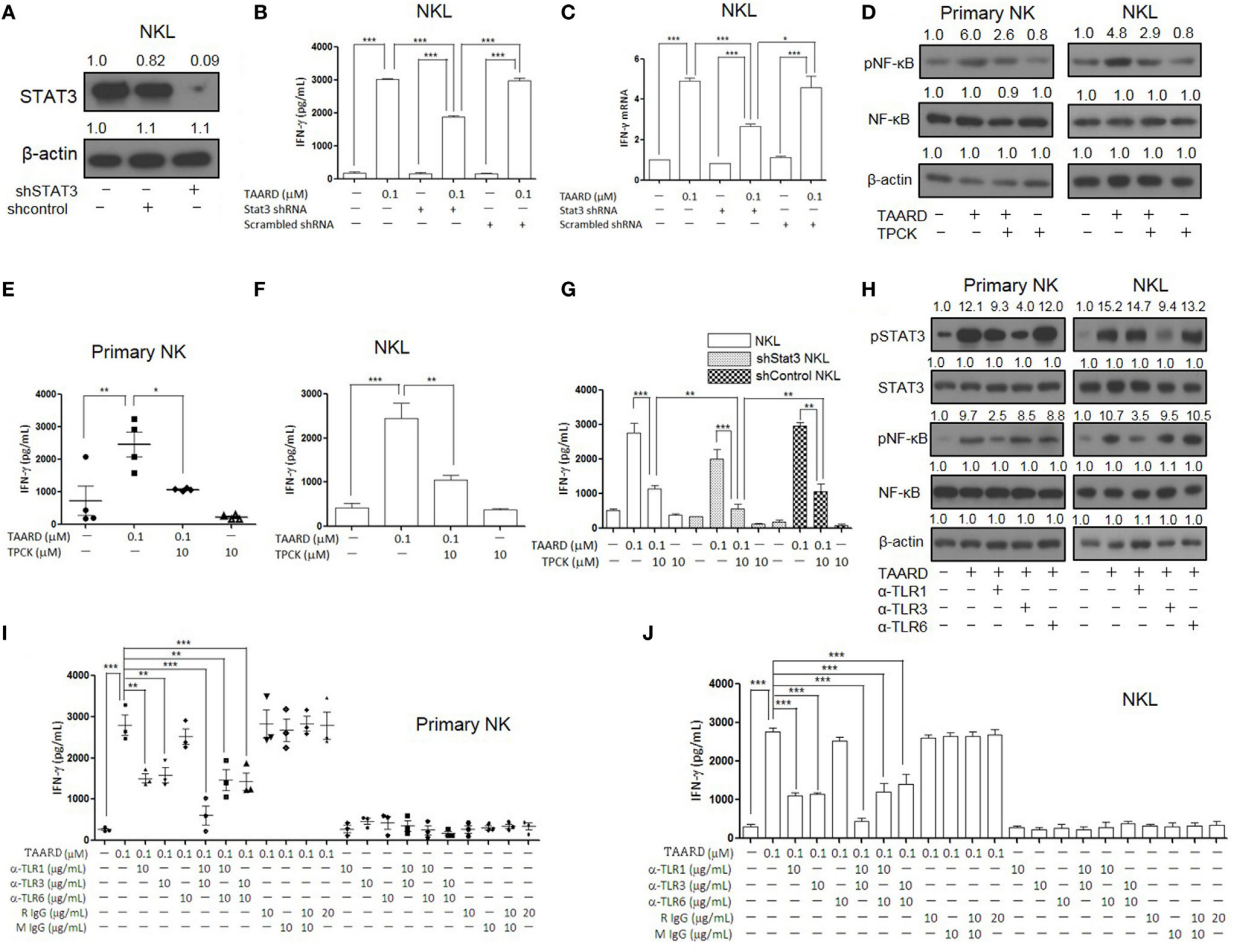
TAARD activates the TLR-1-NF-κB and TLR3-STAT3 signaling pathways in natural killer (NK) cells. NKL cells were infected with concentrated lentivirus of pSIH1-puro-STAT3 shRNA or pSIH1-puro-control shRNA and selected with puromycin. The knockdown of NKL cells were treated with or without TAARD for 18 h. **(A)** STAT3 knockdown was confirmed by immunoblotting. **(B)** Cell-free supernatants were collected for analysis by enzyme-linked immunosorbent assay (ELISA). **(C)** Cell pellets were harvested for real-time RT-PCR. **(D–F)** Primary human NK cells and NKL cells were pretreated with the NF-κB inhibitor tosyl-l-phenylalaninechloromethyl ketone (TPCK) (10 µM) and then exposed to TAARD for 18 h. NF-κB phosphorylation inhibition was confirmed by immunoblotting **(D)** and the supernatants from primary NK **(E)** and NKL **(F)** cells were assayed for interferon (IFN)-γ secretion. **(G)** STAT3-knockdown NKL cells were pretreated with TPCK and then exposed to TAARD for 18 h. The supernatants were collected and analyzed by ELISA. **(H–J)** Human NK cells were pretreated with 10 µg/mL of a non-specific rat or mouse IgG or neutralizing antibodies against TLR1, TLR3, TLR6, or their different combinations for 1 h. Cells were then treated with 0.1 µM of TAARD for an additional 18 h. Supernatants were assayed for IFN-γ secretion and cells were harvested and lysed for immunoblotting of phosphorylated NF-κB and STAT3. The data represent plots of three donors with similar results. Data from one of three independent experiments with similar results are shown. *p < 0.05, **p < 0.01, and ***p < 0.001 denote statistical comparison between the two marked treatment groups.

Next, we used a loss-of-function approach to further confirm the involvement of TLR1-NF-κB and TLR3-STAT3 signaling pathways in TAARD-induced IFN-γ expression in NK cells. We performed a knockdown of *STAT3* using concentrated lentivirus shRNA. Immunoblotting showed that the expression of STAT3 protein decreased significantly in the NKL cell line after shRNA knockdown compared to the negative controls (Figure 5A). This confirmed that our shRNA was functional. We found that IFN-γ secretion decreased notably in STAT3 knock-down NKL cells treated with TAARD when compared to scramble shRNA transduced NKL cells treated with TAARD (Figure 5B). Similar data were observed at the *IFNG* mRNA level (Figure 5C). However, IFN-γ secretion and its mRNA expression levels in STAT3-knockdown NKL cells were still higher than the untreated group, suggesting that TAARD induces IFN-γ secretion only partially through the STAT3 signaling pathway, consistent with our data showing that TAARD stimulates both STAT3 and NF-κB signaling (Figures 3 and 4). Thus, we next pretreated primary NK and NKL cells with the NF-κB inhibitor, TPCK, to explore the involvement of NF-κBp65 in TAARD-mediated IFN-γ expression. We found that TPCK partially inhibited TAARD-mediated phosphorylation of NF-κB (Figure 5D), which in turn resulted in partial suppression of IFN-γ secretion in both primary human NK and NKL cells (Figures 5E,F). Next, we observed that inhibition of both STAT3 signaling by shRNA and NF-κB signaling by TPCK resulted in significantly more IFN-γ suppression when compared to inhibition of either of the signaling pathways alone (Figure 5G), confirming that both signaling pathways are involved in mediating TAARD to induce NK cells to produce IFN-γ.

To further explore the involvement of TLRs in TAARD-mediated IFN-γ secretion in NK cells, we utilized the blocking antibodies of TLR1, TLR3, and TLR6 to neutralize the receptors in primary human NK and NKL cells. The immunoblotting data demonstrate that the α-TLR1 blocking antibody decreased TAARD-induced phosphorylation of NF-κB and the α-TLR3 blocking antibody decreased TAARD-induced phosphorylation of STAT3 (Figure 5H). Meanwhile, α-TLR6 did not produce a noticeable effect on either NF-κB or STAT3 phosphorylation. We also found that blockade of TLR1 or TLR3 alone significantly reduced TAARD-induced IFN-γ secretion, whereas blockade of TLR6 had no significant effect in both primary NK cells (Figure 5I) and the NKL cell line (Figure 5J). Moreover, combined blockade of TLR1 and TLR3 greatly reduced TAARD-enhanced IFN-γ secretion to levels lower than those seen with blockade of either TLR1 or TLR3 in both primary NK cells (Figure 5I) and the NKL cell line (Figure 5J). Next, we detected whether TAARD affected mRNA expression levels of all TLR genes involved. No obvious changes were observed in the expression levels of TLR1, TLR3, or TLR6 after treatment with TAARD alone in primary human NK and NKL cells (Figures S6A, B in Supplementary Material). This indicated that TAARD does not affect mRNA expression levels of TLRs themselves, but plays a role in activating the downstream targets of TLRs. Our results confirmed that TAARD induced IFN-γ expression through TLR1-NF-κB and TLR3-STAT3 signaling pathways in NK cells.

## Discussion

**Figure 6:**
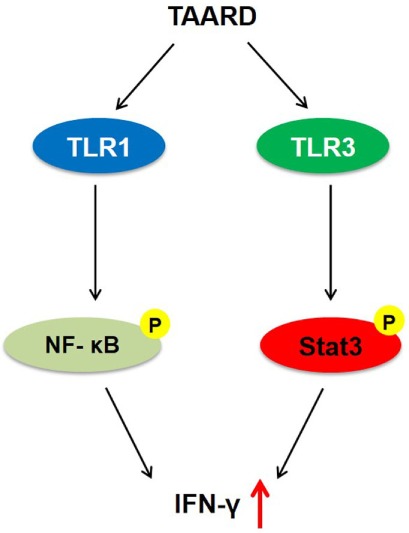
A proposed model by which TAARD stimulates interferon-γ secretion in human natural killer cells *via* TLR1-NF-κB and TLR3–STAT3 signaling pathways.

In our previous study, by screening compounds isolated from plants, we found that a natural product isolated from plants of the *Phyllanthus* genus (e.g., *P. poilanei*), PL-C, induced IFN-γ production by human NK cells *via* activation of NF-κB p65 (16). Purification of PL-C from plants is time-consuming, costly, and limited by material availability. Thus, we performed a *de novo* synthesis of PL-C and successfully generated the disaccharide derivative of diphyllin, TAARD, by connecting a 2,3,4-tri-*O*-acetyl-α-d-arabinose group to a 4-hydroxyl group of 2,3-*O*-isopropylidene-α-l-rhamnose linked directly to a diphyllin unit. We showed that TAARD notably induces human NK cells to produce IFN-γ *via* not only the TLR1-NF-κB signaling pathway but also the TLR3–STAT3 signaling pathway (Figure 6). In addition, TAARD has little adverse effects on human NK cells, indicating that this compound can possibly present a safe alternative to existing therapies, as IFN-γ is a cytokine that plays a critical role in the immune system to fight microbial infections and protect against tumor development (30, 31). Studies have shown that IFN-γ can inhibit the growth of a number of different human tumors *in vitro* (32) and *in vivo* (33).

Although there have been studies involving the effect of natural products on immune cells such as NK cells (34–36), there are limited reports concerning the mechanism behind how natural products and/or their derivatives of medicinal plants act on human immune cells. Our study not only demonstrated that TAARD induces IFN-γ production but also unraveled the underlining mechanism of action. Our characterization supports that both the TLR1-NF-κB and the TLR3–STAT3 signaling pathways mediate TAARD to activate NK cells. Both NF-κB and STAT signaling are important signaling pathways that regulate cytokine gene expression (37). Our characterizations were in line with previous findings. Up to present, at least three NF-κB sites have been characterized, including region −786 to −776 of the NF-κB binding site (KBB site), region −162 to −154 of the CD28 response element-like site (CD28RE) in the 5′ flanking region, and position + 459 to + 470 of the c-Rel binding site (C3) in the first intron (38–40). In addition, regions within the first intron have been identified that are able to bind recombinant members of the STAT family in a cooperative fashion in response to IL-12 treatment (41). The STAT family consists of seven mammalian members (STAT1, STAT2, STAT3, STAT4, STAT5a, STAT5b, and STAT6) that are genetically localized to three chromosomal regions. The STAT proteins are unique among transcription factors in that they contain an SH2 (src-homology 2) phosphotyrosine-binding domain (42). The potential of the SH2 domain to interact with a number of signaling proteins allows the STAT proteins to interfere with multiple signaling pathways. Upon activation by tyrosine kinases, members of the STAT family form stable dimers that are able to rapidly translocate to the nucleus and bind DNA (43). The STAT DNA-binding domain has a striking structural similarity to the NF-κB DNA-binding domain, suggesting a common evolutionary origin (44). Further studies revealed that optimal IFN-γ gene expression was dependent upon the synergism of both promoter and intronic enhancer regions (45).

Interestingly, we found that the upstream player of both NF-κB and STAT3 signaling mediating the effect of TAARD is TLR signaling, although different TLR family members are involved, i.e., TLR1 for NF-κB and TLR3 for STAT3 signaling. TLR signaling is important in mediating the effects of natural products and regulating immune responses (46). The TLR family is a class of proteins that play a key role in the innate immune system and has 10 members (TLR1-TLR10) in humans (47). TLR signaling consists of at least two distinct pathways: a MyD88-dependent pathway that leads to the production of inflammatory cytokines and a MyD88-independent pathway associated with the stimulation of IFN-β and the maturation of dendritic cells (48). The MyD88-dependent pathway is common to all TLRs, except TLR3 (49). Upon activation by pathogen-associated microbial patterns or danger-associated molecular patterns, TLRs hetero- or homodimerize and induce the recruitment of adaptor proteins *via* the cytoplasmic TIR domain. It is reported that TLR1 and TLR6 both form heterodimers with TLR2 and share a 56% sequence identity (50). In NK cells, TLR1, TLR2, TLR3, TLR5, and TLR6 are primarily expressed, whereas TLR4, TLR7, TLR8, and TLR9 have low expression (51). In our previous study, we found that TLR1, TLR3, and TLR6 are the main TLRs expressed in human NK cells (18). In this study, we found that TAARD induces the signaling pathways of TLR1 and TLR3, but not TLR6. TLR1 is a membrane-associated sensor expressed on the cell surface while TLR3 is localized in the endosomal membrane and forms a homodimer (52). However, how TAARD interacts with and triggers the downstream signaling of TLR1 and TLR3 remains to be determined.

Both PL-C (16) and its synthetic disaccharide derivative, TAARD, can induce NK cell IFN-γ production through induction of TLR1-NF-κB signaling. However, these structurally different agents were found to have some unique characteristics. TAARD triggers TLR3-STAT3 signaling, while there is no evidence showing that PL-C does the same. Thus, these data suggest that while the structure determines the function of a compound, parent compounds and their derivatives with similar structures can target different, but related signaling pathways to achieve similar outcomes of immune cell regulation.

Previous *in vitro* studies showed that the combination of IL-12 and IL-18 or IL-15 strongly induces human NK cells to produce IFN-γ, especially compared to individual cytokines (53). The IL-15 superagonist complex with IL-15Rα, named ALT-803, is being actively tested in the clinic for the treatment of cancer (54). These cytokines have also been shown to induce memory-like NK cells of mice and humans (55, 56). It has also been shown that adoptive transfer of cytokine-induced memory NK cells into acute myeloid leukemia results in proliferation and expansion of these cells, leading to robust responses against leukemia targets (54). It will be interesting to know whether TAARD has the potential for clinical application by activating NK cells in patients and/or inducing memory-like NK cells *ex vivo* prior to adoptive cell transfer.

The present investigation provides us with critical information about the mechanism of how natural products stimulate human NK cells. Both NK cells and IFN-γ are used in the clinic for treatment of cancer and other diseases. Numerous current clinical trials have attempted to use exogenously administered IFN-γ to treat malignancies but have met with disappointing results due to mixed outcomes and the potential for severe side effects (57). Likewise, utilization of cytokines, including IL-12 and IL-2, to increase the production of IFN-γ endogenously, also present with their own shortcomings (11–13). Administration of IL-2 to activate NK cells in cancer patients led to varied results, depending on the type of tumor tested and the experimental conditions (13). High-doses of IL-2 are associated with toxic buildup in many organ systems, including the central nervous system, heart, kidneys, and lungs (58). IL-2 signaling is also required to maintain the fitness and homeostasis of immunosuppressive regulatory T cells (59). IL-12 results in minimal response with toxic side effects due to high levels of IFN-γ production that prime NK cells to augment cytotoxic as well as cell-mediated immune responses (60). Thus, it is of paramount importance to find an effective and convenient therapeutic drug to stimulate NK cells. Here, we present data showing that a *de novo*-synthetic compound from a natural product, diphyllin, can induce NK cell secretion of IFN-γ and possibly have anticancer effects while leaving cell growth and apoptosis of normal NK cells intact. Our work demonstrates an alternative approach to invent a novel drug for cancer prevention and can lead the way to further exploration of natural products in areas such as infectious disease and allergy.

Several studies have demonstrated that natural products enhance NK cell function. However, modification of natural products as an alternative approach to induce NK cell activity or immune responses widely and economically is less explored. The present study suggests that this is a feasible and quick approach to generate novel compounds to enhance immune responses, although more studies are needed to test whether the compound will eventually have clinical applications.

In conclusion, our current study presents an alternative approach to generate new compounds based on the structure of natural products to modulate the immune responses of human NK cells. Through this approach, we have generated a novel compound called TAARD that is capable of inducing NK cell IFN-γ production *via* both the TLR1-NF-κB and the TLR3–STAT3 signaling pathways.

## Author Contributions

JY, XH, LSW, AK, ZF, and MC designed experiments. LY, LC, and JY wrote the manuscript and analyzed the data. LY, XG, TL, HW, XJ, YR, and PP performed experiments and analyzed data. JZ helped with statistical design and analysis. JY, MC, and ZF acquired funding. JY supervised the study.

## Conflict of Interest Statement

The authors declare that the research was conducted in the absence of any commercial or financial relationships that could be construed as a potential conflict of interest. The reviewer, TF and the handling Editor declared their shared affiliation.

## Supplementary Material

Figure S1 Molecular structure of TAARD.

Figure S2 Purity of enriched and purified human primary NK cells. Representative flow data from 1 out of 4 donors show the purity of enriched **(A)** and purified **(B)** human NK cells. The purity of enriched and purified NK cells was >70 and >98%, respectively.

Figure S3 TAARD has little adverse effect on primary human NK cells. **(A,B)** Primary human NK cells were treated with different concentrations (0.1, 1, and 10 µM) of TAARD for different time intervals. The cell viability was measured by cell counting **(A)** and the MTS assay **(B)**. **(C,D)** Purified NK cells were treated with different concentrations (0.1, 1, and 10 µM) of TAARD for 18 h. Pellets were collected and incubated with annexin V-fluorescein isothiocyanate plus 7-AAD. The percentage of apoptotic cells were determined by a flow cytometric analysis.

Figure S4 Purity of CD56^bright^ and CD56^dim^ NK cells. Representative flow data from 1 out of 3 donors show the purity of CD56^bright^ **(A)** and CD56^dim^ **(B)** human NK cells.

Figure S5 TAARD does not affect phosphorylation of STAT4 and STAT5 in NK cells. **(A,B)** Purified primary human NK cells and NKL cells were treated with 0.1 µM of either TAARD alone or in combination with 100 ng/mL of IL-12 **(A)** or 100 ng/mL of IL-15 **(B)** for 6 h. Cells were harvested and lysed for immunoblotting using antibodies against STAT4 or STAT5. β-actin was included as the internal control.

Figure S6 Effects of TAARD on the mRNA expression levels of potential target genes. Purified human NK and NKL cells were treated with 0.1 µM of TAARD for 18 h and then cell pellets were harvested to detect mRNA expression levels by real-time RT-PCR. Data shown are the means of three donors.

Figure S7 TAARD enhances NF-κBp65 and STAT3 promoter reporter activities through TLR signaling. **(A)** 293T cells were co-transfected with either pGL3-κB-luc (1 µg) or pGL Basic plasmid and pRL-TK renilla-luciferase control plasmids (5 ng) in the presence or absence of a TLR3 expression plasmid (0.5 µg). **(B)** 293T cells were co-transfected as described in **(A)** but with a TLR6 (0.5 µg) instead of a TLR3 expression plasmid. **(C)** 293T cells were co-transfected with either 4×M67 pTATA TK-Luc or pGL Basic and pRL-TK renilla-luciferase control plasmids (5 ng) in the presence or absence of a TLR1 expression plasmid (0.5 µg). **(D)** NKL cells were co-transfected as described in **(C)** but with a TLR6 expression plasmid (0.5 µg) instead of TLR-1 expression plasmid. Cells were treated and the luciferase activities were measured as described in Figure 5.

## References

[1] Newman DJ Cragg GM. Natural products as sources of new drugs from 1981 to 2014. J Nat Prod (2016) 79:629–61. 10.1021/acs.jnatprod.5b01055 26852623

[2] Butler MS Robertson AA Cooper MA. Natural product and natural product derived drugs in clinical trials. Nat Prod Rep (2014) 31:1612–61. 10.1039/c4np00064a 25204227

[3] Dias DA Urban S Roessner U. A historical overview of natural products in drug discovery. Metabolites (2012) 2:303–36. 10.3390/metabo2020303 24957513 PMC3901206

[4] Li Y Li S Meng X Gan RY Zhang JJ Li HB Dietary natural products for prevention and treatment of breast cancer. Nutrients (2017) 9:E728 10.3390/nu9070728 28698459 PMC5537842

[5] Shanmugam MK Warrier S Kumar AP Sethi G Arfuso F. Potential role of natural compounds as anti-angiogenic agents in cancer. Curr Vasc Pharmacol (2017) 15(6):503–19. 10.2174/1570161115666170713094319 28707601

[6] Yatim KM Lakkis FG. A brief journey through the immune system. Clin J Am Soc Nephrol (2015) 10:1274–81. 10.2215/CJN.10031014 25845377 PMC4491295

[7] Vivier E Tomasello E Baratin M Walzer T Ugolini S. Functions of natural killer cells. Nat Immunol (2008) 9:503–10. 10.1038/ni1582 18425107

[8] Caligiuri MA. Human natural killer cells. Blood (2008) 112:461–9. 10.1182/blood-2007-09-077438 18650461 PMC2481557

[9] Colucci F Caligiuri MA Di Santo JP. What does it take to make a natural killer? Nat Rev Immunol (2003) 3:413–25. 10.1038/nri1088 12766763

[10] Dunn GP Koebel CM Schreiber RD. Interferons, immunity and cancer immunoediting. Nat Rev Immunol (2006) 6:836–48. 10.1038/nri1961 17063185

[11] Strengell M Matikainen S Siren J Lehtonen A Foster D Julkunen I IL-21 in synergy with IL-15 or IL-18 enhances IFN-gamma production in human NK and T cells. J Immunol (2003) 170:5464–9. 10.4049/jimmunol.170.11.5464 12759422

[12] Salem ML Gillanders WE Kadima AN El-Naggar S Rubinstein MP Demcheva M Review: novel nonviral delivery approaches for interleukin-12 protein and gene systems: curbing toxicity and enhancing adjuvant activity. J Interferon Cytokine Res (2006) 26:593–608. 10.1089/jir.2006.26.593 16978064

[13] Gowda A Ramanunni A Cheney C Rozewski D Kindsvogel W Lehman A Differential effects of IL-2 and IL-21 on expansion of the CD4+ CD25+ Foxp3+ T regulatory cells with redundant roles in natural killer cell mediated antibody dependent cellular cytotoxicity in chronic lymphocytic leukemia. MAbs (2010) 2:35–41. 10.4161/mabs.2.1.10561 20081380 PMC2828576

[14] Ren Y Yuan C Deng Y Kanagasabai R Ninh TN Tu VT Cytotoxic and natural killer cell stimulatory constituents of *Phyllanthus songboiensis*. Phytochemistry (2015) 111:132–40. 10.1016/j.phytochem.2014.12.014 25596805 PMC4333069

[15] Ren Y Lantvit DD Deng Y Kanagasabai R Gallucci JC Ninh TN Potent cytotoxic arylnaphthalene lignan lactones from *Phyllanthus poilanei*. J Nat Prod (2014) 77:1494–504. 10.1021/np5002785 24937209 PMC4073661

[16] Deng Y Chu J Ren Y Fan Z Ji X Mundy-Bosse B The natural product phyllanthusmin C enhances IFN-gamma production by human NK cells through upregulation of TLR-mediated NF-kappaB signaling. J Immunol (2014) 193:2994–3002. 10.4049/jimmunol.1302600 25122922 PMC4162489

[17] Zhang HJ Rumschlag-Booms E Guan YF Liu KL Wang DY Li WF Anti-HIV diphyllin glycosides from *Justicia gendarussa*. Phytochemistry (2017) 136:94–100. 10.1016/j.phytochem.2017.01.005 28110956

[18] He S Chu J Wu LC Mao H Peng Y Alvarez-Breckenridge CA MicroRNAs activate natural killer cells through toll-like receptor signaling. Blood (2013) 121:4663–71. 10.1182/blood-2012-07-441360 23580661 PMC3674667

[19] Sun R Zhang Y Lv Q Liu B Jin M Zhang W Toll-like receptor 3 (TLR3) induces apoptosis via death receptors and mitochondria by up-regulating the transactivating p63 isoform alpha (TAP63alpha). J Biol Chem (2011) 286:15918–28. 10.1074/jbc.M110.178798 21367858 PMC3091201

[20] Oosting M Ter Hofstede H Sturm P Adema GJ Kullberg BJ Van Der Meer JW TLR1/TLR2 heterodimers play an important role in the recognition of *Borrelia spirochetes*. PLoS One (2011) 6:e25998. 10.1371/journal.pone.0025998 21998742 PMC3187844

[21] Yu J Wei M Becknell B Trotta R Liu S Boyd Z Pro- and antiinflammatory cytokine signaling: reciprocal antagonism regulates interferon-gamma production by human natural killer cells. Immunity (2006) 24:575–90. 10.1016/j.immuni.2006.03.016 16713975

[22] Yu J Mao HC Wei M Hughes T Zhang J Park IK CD94 surface density identifies a functional intermediary between the CD56bright and CD56dim human NK-cell subsets. Blood (2010) 115:274–81. 10.1182/blood-2009-04-215491 19897577 PMC2808153

[23] Trotta R Ciarlariello D Dal Col J Mao H Chen L Briercheck E The PP2A inhibitor SET regulates granzyme B expression in human natural killer cells. Blood (2011) 117:2378–84. 10.1182/blood-2010-05-285130 21156847 PMC3062407

[24] Besser D Bromberg JF Darnell JE Jr Hanafusa H. A single amino acid substitution in the v-Eyk intracellular domain results in activation of Stat3 and enhances cellular transformation. Mol Cell Biol (1999) 19:1401–9. 10.1128/MCB.19.2.1401 9891073 PMC116068

[25] Huang S Sinicrope FA. Sorafenib inhibits STAT3 activation to enhance TRAIL-mediated apoptosis in human pancreatic cancer cells. Mol Cancer Ther (2010) 9:742–50. 10.1158/1535-7163.MCT-09-1004 20197401 PMC3281304

[26] Moretta L Dissecting CD56dim human NK cells. Blood (2010) 116:3689–91. 10.1182/blood-2010-09-303057 21071612

[27] Mogensen TH. Pathogen recognition and inflammatory signaling in innate immune defenses. Clin Microbiol Rev (2009) 22:240–73. 10.1128/CMR.00046-08 19366914 PMC2668232

[28] Hornung V Rothenfusser S Britsch S Krug A Jahrsdorfer B Giese T Quantitative expression of toll-like receptor 1-10 mRNA in cellular subsets of human peripheral blood mononuclear cells and sensitivity to CpG oligodeoxynucleotides. J Immunol (2002) 168:4531–7. 10.4049/jimmunol.168.9.4531 11970999

[29] Makela SM Strengell M Pietila TE Osterlund P Julkunen I. Multiple signaling pathways contribute to synergistic TLR ligand-dependent cytokine gene expression in human monocyte-derived macrophages and dendritic cells. J Leukoc Biol (2009) 85:664–72. 10.1189/jlb.0808503 19164128

[30] Ikeda H Old LJ Schreiber RD. The roles of IFN gamma in protection against tumor development and cancer immunoediting. Cytokine Growth Factor Rev (2002) 13:95–109. 10.1016/S1359-6101(01)00038-7 11900986

[31] Baer C Squadrito ML Laoui D Thompson D Hansen SK Kiialainen A Suppression of microRNA activity amplifies IFN-gamma-induced macrophage activation and promotes anti-tumour immunity. Nat Cell Biol (2016) 18:790–802. 10.1038/ncb3371 27295554

[32] Aune TM Pogue SL. Inhibition of tumor cell growth by interferon-gamma is mediated by two distinct mechanisms dependent upon oxygen tension: induction of tryptophan degradation and depletion of intracellular nicotinamide adenine dinucleotide. J Clin Invest (1989) 84:863–75. 10.1172/JCI114247 2503544 PMC329730

[33] Ko JH Jung BG Park YS Lee BJ. Inhibitory effects of interferon-gamma plasmid DNA on DMBA-TPA induced mouse skin carcinogenesis. Cancer Gene Ther (2011) 18:646–54. 10.1038/cgt.2011.36 21799530

[34] Armeanu S Bitzer M Lauer UM Venturelli S Pathil A Krusch M Natural killer cell-mediated lysis of hepatoma cells via specific induction of NKG2D ligands by the histone deacetylase inhibitor sodium valproate. Cancer Res (2005) 65:6321–9. 10.1158/0008-5472.CAN-04-4252 16024634

[35] Kim HR Kim K Lee KH Kim SJ Kim J. Inhibition of casein kinase 2 enhances the death ligand- and natural killer cell-induced hepatocellular carcinoma cell death. Clin Exp Immunol (2008) 152:336–44. 10.1111/j.1365-2249.2008.03622.x 18336591 PMC2384109

[36] Lu CC Chen JK. Resveratrol enhances perforin expression and NK cell cytotoxicity through NKG2D-dependent pathways. J Cell Physiol (2010) 223:343–51. 10.1002/jcp.22043 20082299

[37] Fan Y Mao R Yang J NF-kappaB and STAT3 signaling pathways collaboratively link inflammation to cancer. Protein Cell (2013) 4:176–85. 10.1007/s13238-013-2084-3 23483479 PMC4875500

[38] Sica A Tan TH Rice N Kretzschmar M Ghosh P Young HA. The c-rel protooncogene product c-Rel but not NF-kappa B binds to the intronic region of the human interferon-gamma gene at a site related to an interferon-stimulable response element. Proc Natl Acad Sci U S A (1992) 89:1740–4. 10.1073/pnas.89.5.1740 1542667 PMC48528

[39] Lai JH Horvath G Subleski J Bruder J Ghosh P Tan TH. RelA is a potent transcriptional activator of the CD28 response element within the interleukin 2 promoter. Mol Cell Biol (1995) 15:4260–71. 10.1128/MCB.15.8.4260 7623820 PMC230665

[40] Young HA. Regulation of interferon-gamma gene expression. J Interferon Cytokine Res (1996) 16:563–8. 10.1089/jir.1996.16.563 8877725

[41] Gonsky R Deem RL Bream J Young HA Targan SR. Enhancer role of STAT5 in CD2 activation of IFN-gamma gene expression. J Immunol (2004) 173:6241–7. 10.4049/jimmunol.173.10.6241 15528362

[42] Rawlings JS Rosler KM Harrison DA The JAK/STAT signaling pathway. J Cell Sci (2004) 117:1281–3. 10.1242/jcs.00963 15020666

[43] Levy DE Darnell JE Jr. Stats: transcriptional control and biological impact. Nat Rev Mol Cell Biol (2002) 3:651–62. 10.1038/nrm909 12209125

[44] Yu Z Kone BC. The STAT3 DNA-binding domain mediates interaction with NF-kappaB p65 and inducible nitric oxide synthase transrepression in mesangial cells. J Am Soc Nephrol (2004) 15:585–91. 10.1097/01.ASN.0000114556.19556.F9 14978160

[45] Sica A Dorman L Viggiano V Cippitelli M Ghosh P Rice N Interaction of NF-kappaB and NFAT with the interferon-gamma promoter. J Biol Chem (1997) 272:30412–20. 10.1074/jbc.272.48.30412 9374532

[46] Chen L Yu J. Modulation of toll-like receptor signaling in innate immunity by natural products. Int Immunopharmacol (2016) 37:65–70. 10.1016/j.intimp.2016.02.005 26899347 PMC4916003

[47] Takeda K Akira S. Toll-like receptors in innate immunity. Int Immunol (2005) 17:1–14. 10.1093/intimm/dxh186 15585605

[48] Warner N Nunez G MyD88: a critical adaptor protein in innate immunity signal transduction. J Immunol (2013) 190:3–4. 10.4049/jimmunol.1203103 23264668

[49] Broad A Kirby JA Jones DE Applied Immunology and Transplantation Research Group. Toll-like receptor interactions: tolerance of MyD88-dependent cytokines but enhancement of MyD88-independent interferon-beta production. Immunology (2007) 120:103–11. 10.1111/j.1365-2567.2006.02485.x 17034424 PMC2265871

[50] Jin MS Kim SE Heo JY Lee ME Kim HM Paik SG Crystal structure of the TLR1-TLR2 heterodimer induced by binding of a tri-acylated lipopeptide. Cell (2007) 130:1071–82. 10.1016/j.cell.2007.09.008 17889651

[51] Adib-Conquy M Scott-Algara D Cavaillon JM Souza-Fonseca-Guimaraes F. TLR-mediated activation of NK cells and their role in bacterial/viral immune responses in mammals. Immunol Cell Biol (2014) 92:256–62. 10.1038/icb.2013.99 24366517

[52] Choe J Kelker MS Wilson IA. Crystal structure of human toll-like receptor 3 (TLR3) ectodomain. Science (2005) 309:581–5. 10.1126/science.1115253 15961631

[53] Fehniger TA Shah MH Turner MJ Vandeusen JB Whitman SP Cooper MA Differential cytokine and chemokine gene expression by human NK cells following activation with IL-18 or IL-15 in combination with IL-12: implications for the innate immune response. J Immunol (1999) 162:4511–20. 10201989

[54] Romee R Cooley S Berrien-Elliott MM Westervelt P Verneris MR Wagner JE First-in-human phase 1 clinical study of the IL-15 superagonist complex ALT-803 to treat relapse after transplantation. Blood (2018) 131:2515–27. 10.1182/blood-2017-12-823757 29463563 PMC5992862

[55] Cooper MA Elliott JM Keyel PA Yang L Carrero JA Yokoyama WM. Cytokine-induced memory-like natural killer cells. Proc Natl Acad Sci U S A (2009) 106:1915–9. 10.1073/pnas.0813192106 19181844 PMC2644138

[56] Romee R Schneider SE Leong JW Chase JM Keppel CR Sullivan RP Cytokine activation induces human memory-like NK cells. Blood (2012) 120:4751–60. 10.1182/blood-2012-04-419283 22983442 PMC3520618

[57] Zaidi MR Merlino G The two faces of interferon-gamma in cancer. Clin Cancer Res (2011) 17:6118–24. 10.1158/1078-0432.CCR-11-0482 21705455 PMC3186825

[58] Schwartz RN Stover L Dutcher J. Managing toxicities of high-dose interleukin-2. Oncology (Williston Park) (2002) 16:11–20. 12469935

[59] Fontenot JD Rasmussen JP Gavin MA Rudensky AY. A function for interleukin 2 in Foxp3-expressing regulatory T cells. Nat Immunol (2005) 6:1142–51. 10.1038/ni1263 16227984

[60] Colombo MP Trinchieri G. Interleukin-12 in anti-tumor immunity and immunotherapy. Cytokine Growth Factor Rev (2002) 13:155–68. 10.1016/S1359-6101(01)00032-6 11900991

