# Supplementary figures and images for: A Synthetic Disaccharide Derivative of Diphyllin, TAARD, Activates Human Natural Killer Cells to Secrete Interferon-Gamma via Toll-Like Receptor-Mediated NF-κB and STAT3 Signaling Pathways
Source: Front Immunol. 2018 Jul 18;9:1509. doi: 10.3389/fimmu.2018.01509 (PMC6058043; doi:10.3389/fimmu.2018.01509)

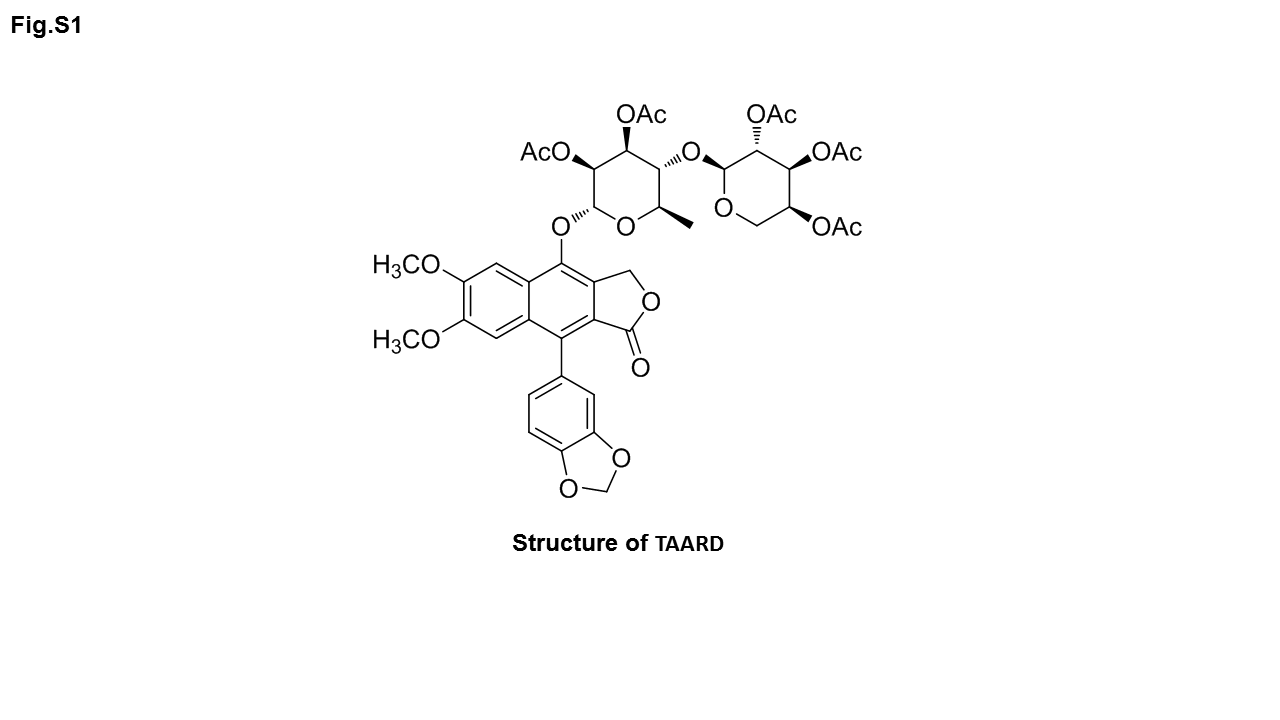

Supplement: Figure S1 — Molecular structure of TAARD. [file image_1.tif]

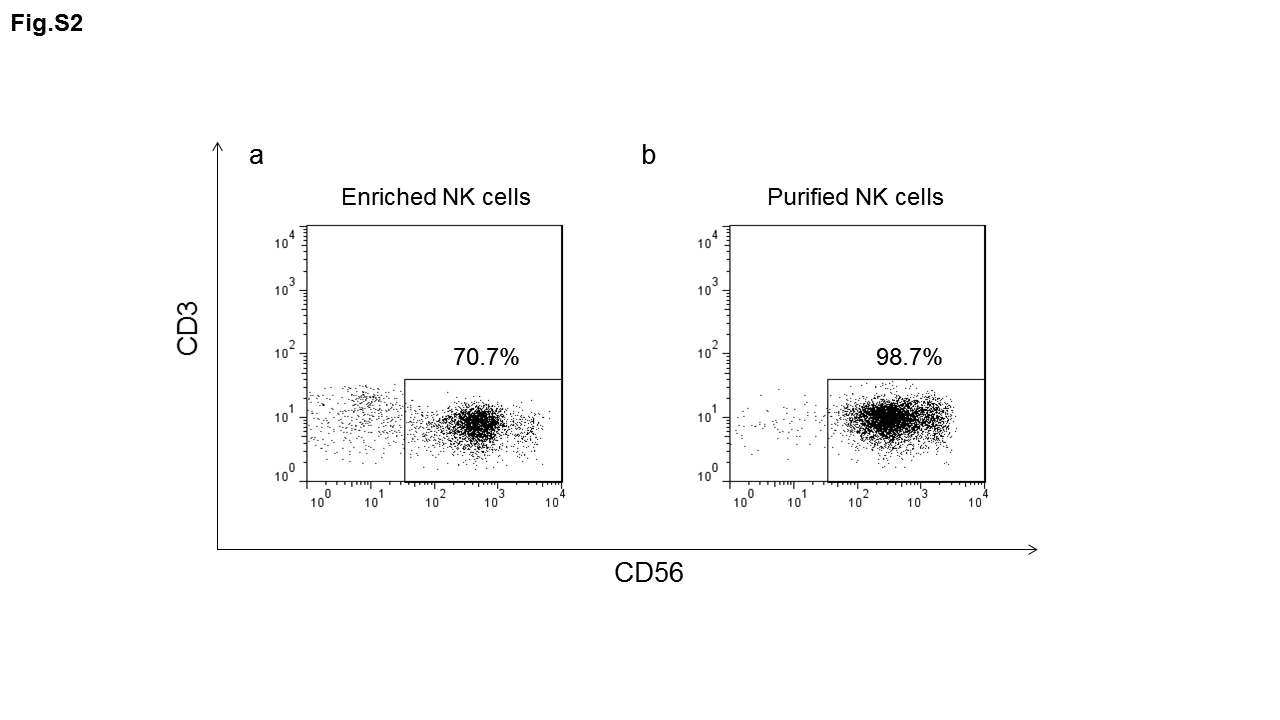

Supplement: Figure S2 — Purity of enriched and purified human primary NK cells. Representative flow data from 1 out of 4 donors show the purity of enriched (A) and purified (B) human NK cells. The purity of enriched and purified NK cells was >70 and >98%, respectively. [file image_2.tif]

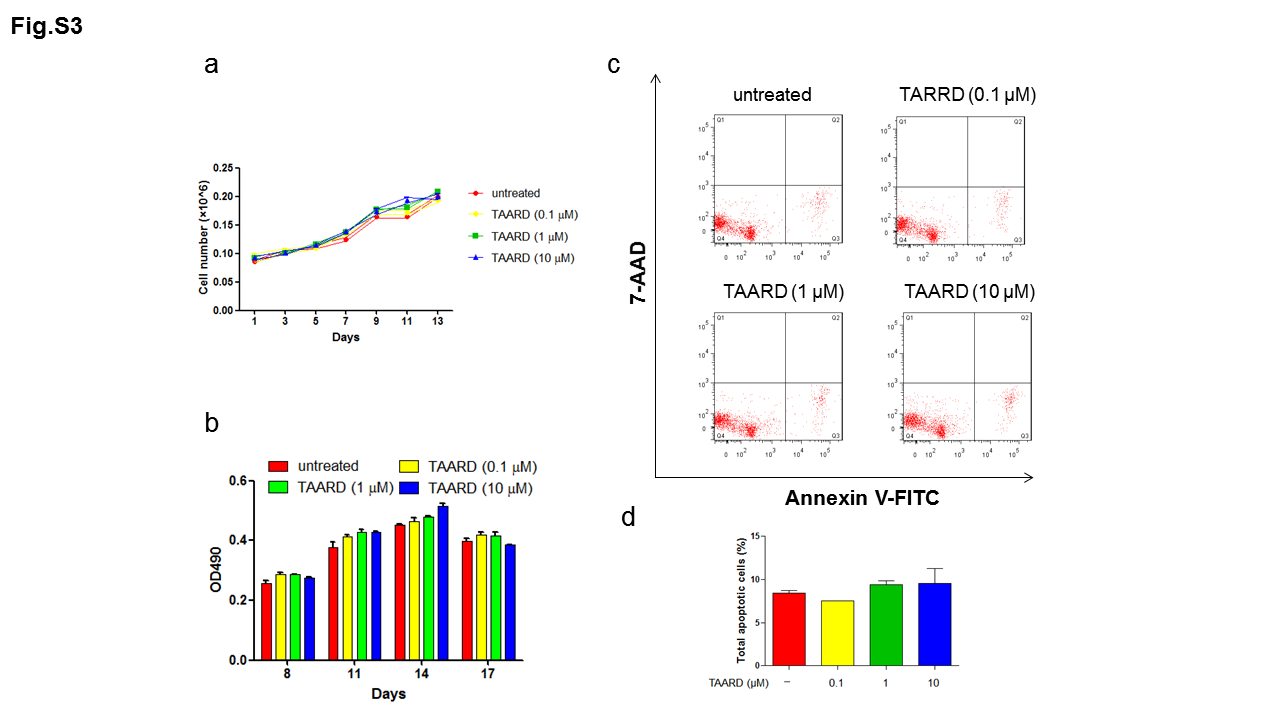

Supplement: Figure S3 — TAARD has little adverse effect on primary human NK cells. (A,B) Primary human NK cells were treated with different concentrations (0.1, 1, and 10 µM) of TAARD for different time intervals. The cell viability was measured by cell counting (A) and the MTS assay (B). (C,D) Purified NK cells were treated with different concentrations (0.1, 1, and 10 µM) of TAARD for 18 h. Pellets were collected and incubated with annexin V-fluorescein isothiocyanate plus 7-AAD. The percentage of apoptotic cells were determined by a flow cytometric analysis. [file image_3.tif]

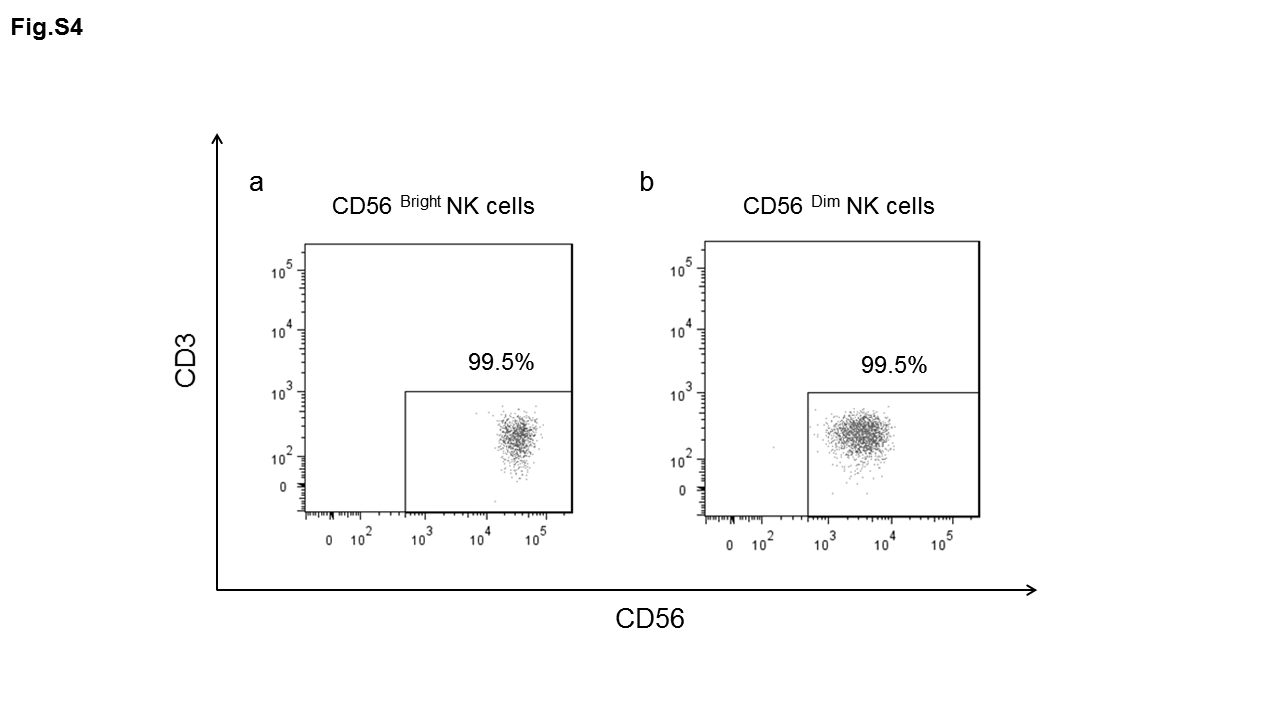

Supplement: Figure S4 — Purity of CD56bright and CD56dim NK cells. Representative flow data from 1 out of 3 donors show the purity of CD56bright (A) and CD56dim (B) human NK cells. [file image_4.tif]

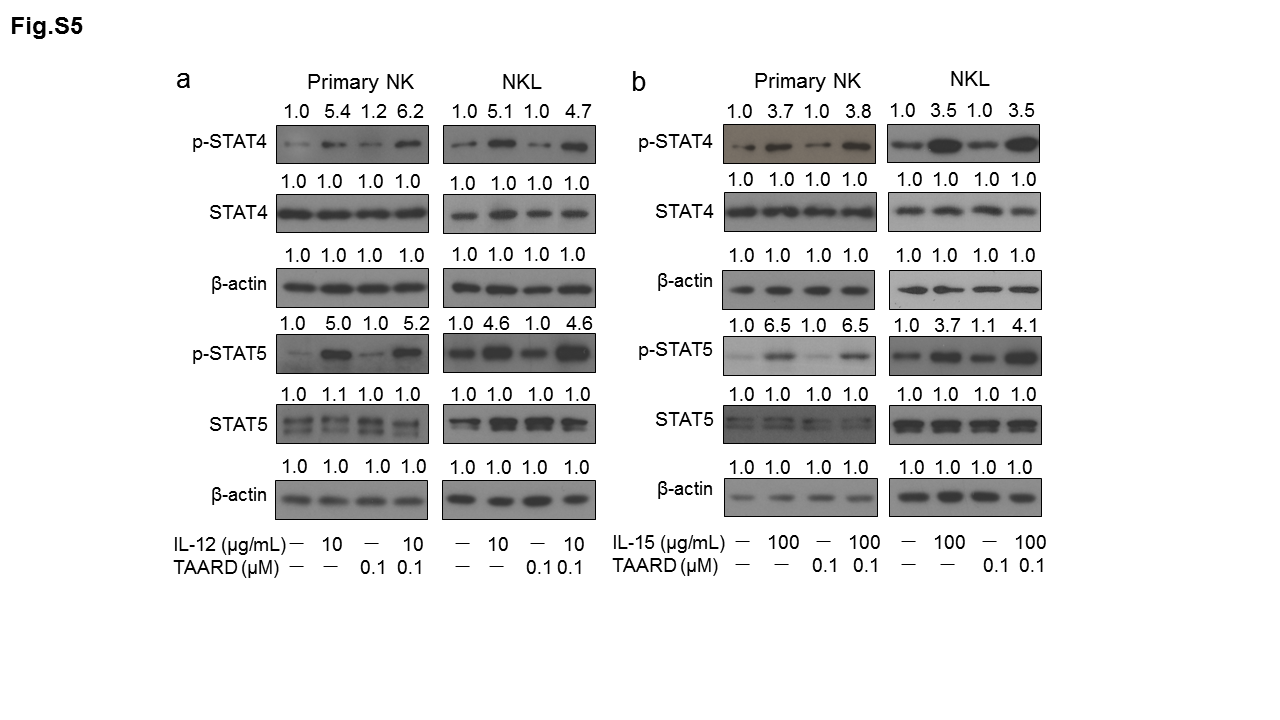

Supplement: Figure S5 — TAARD does not affect phosphorylation of STAT4 and STAT5 in NK cells. (A,B) Purified primary human NK cells and NKL cells were treated with 0.1 µM of either TAARD alone or in combination with 100 ng/mL of IL-12 (A) or 100 ng/mL of IL-15 (B) for 6 h. Cells were harvested and lysed for immunoblotting using antibodies against STAT4 or STAT5. β-actin was included as the internal control. [file image_5.tif]

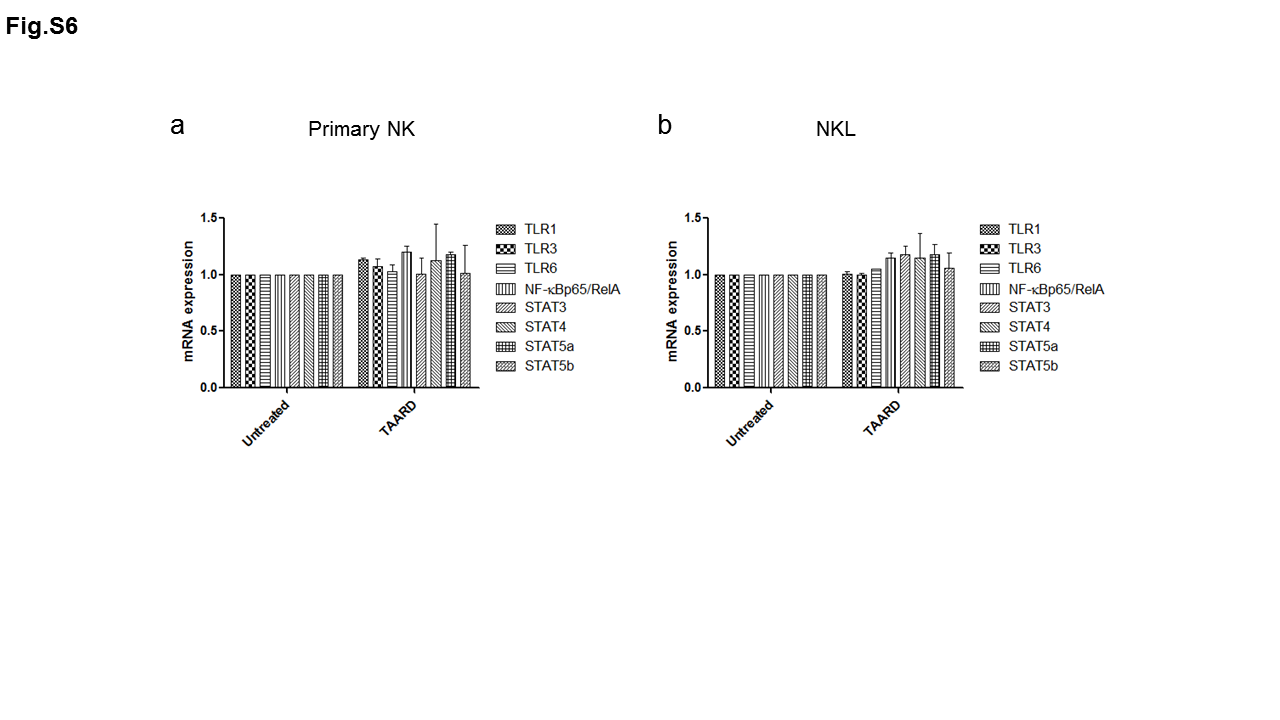

Supplement: Figure S6 — Effects of TAARD on the mRNA expression levels of potential target genes. Purified human NK and NKL cells were treated with 0.1 µM of TAARD for 18 h and then cell pellets were harvested to detect mRNA expression levels by real-time RT-PCR. Data shown are the means of three donors. [file image_6.tif]

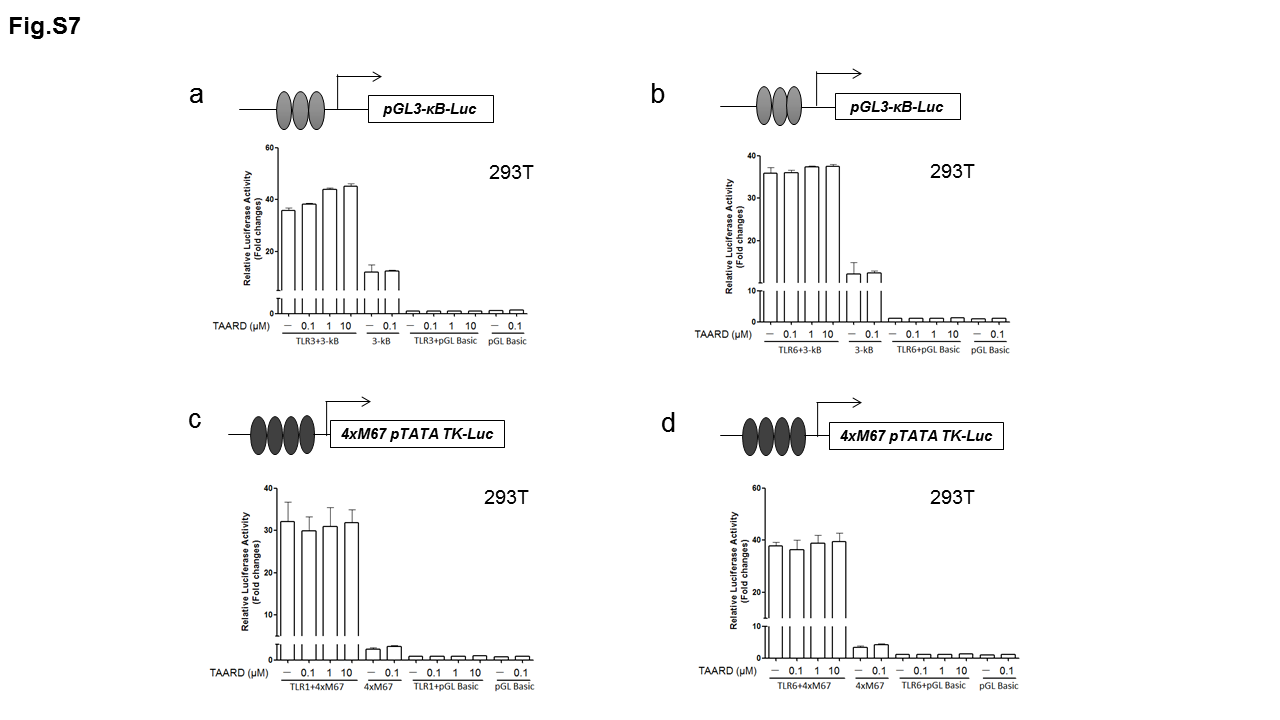

Supplement: Figure S7 — TAARD enhances NF-κBp65 and STAT3 promoter reporter activities through TLR signaling. (A) 293T cells were co-transfected with either pGL3-κB-luc (1 µg) or pGL Basic plasmid and pRL-TK renilla-luciferase control plasmids (5 ng) in the presence or absence of a TLR3 expression plasmid (0.5 µg). (B) 293T cells were co-transfected as described in (A) but with a TLR6 (0.5 µg) instead of a TLR3 expression plasmid. (C) 293T cells were co-transfected with either 4×M67 pTATA TK-Luc or pGL Basic and pRL-TK renilla-luciferase control plasmids (5 ng) in the presence or absence of a TLR1 expression plasmid (0.5 µg). (D) NKL cells were co-transfected as described in (C) but with a TLR6 expression plasmid (0.5 µg) instead of TLR-1 expression plasmid. Cells were treated and the luciferase activities were measured as described in Figure 5. [file image_7.tif]

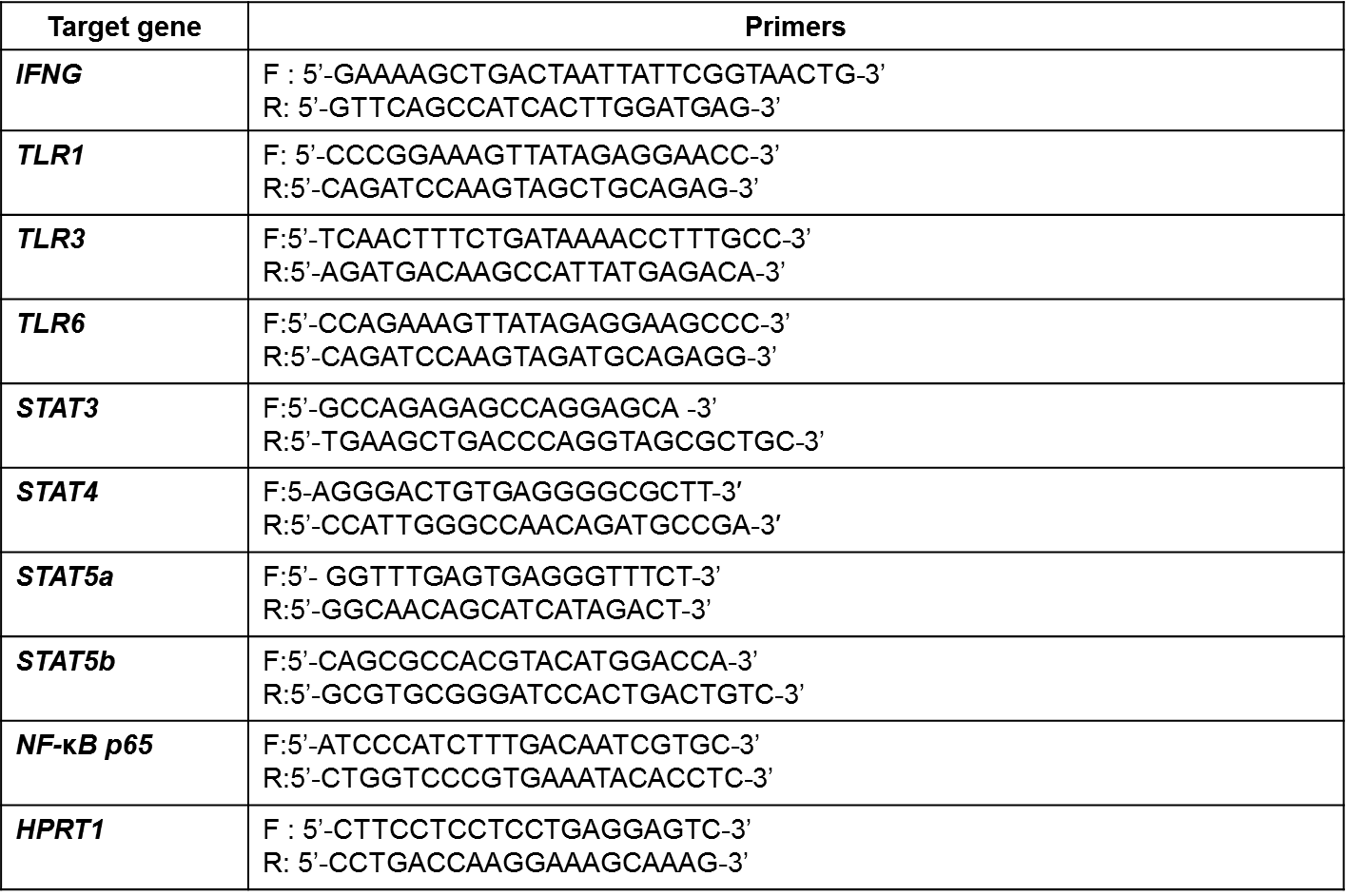


**Supplementary Table 1: Primers for Real Time (RT) PCR**

Supplement: Supplementary file 8 [file table_1.docx]
